# Exogenous Nicotinamide Adenine Dinucleotide Induces Resistance to Citrus Canker in Citrus

**DOI:** 10.3389/fpls.2018.01472

**Published:** 2018-10-09

**Authors:** Fernando M. Alferez, Kayla M. Gerberich, Jian-Liang Li, Yanping Zhang, James H. Graham, Zhonglin Mou

**Affiliations:** ^1^Citrus Research and Education Center, University of Florida, Lake Alfred, FL, United States; ^2^Southwest Florida Research and Education Center, University of Florida, Immokalee, FL, United States; ^3^National Institute of Environmental Health Sciences, National Institutes of Health, Durham, NC, United States; ^4^Interdisciplinary Center for Biotechnology Research, University of Florida, Gainesville, FL, United States; ^5^Department of Microbiology and Cell Science, University of Florida, Gainesville, FL, United States

**Keywords:** extracellular NAD, disease resistance, transcriptional changes, citrus canker, *Xanthomonas citri* subsp. *citri*, defense gene, *Citrus sinensis*

## Abstract

Nicotinamide adenine dinucleotide (NAD) is a universal electron carrier that participates in important intracellular metabolic reactions and signaling events. Interestingly, emerging evidence in animals indicates that cellular NAD can be actively or passively released into the extracellular space, where it is processed or perceived by ectoenzymes or cell-surface receptors. We have recently shown in *Arabidopsis thaliana* that exogenous NAD induces defense responses, that pathogen infection leads to release of NAD into the extracellular space at concentrations sufficient for defense activation, and that depletion of extracellular NAD (eNAD) by transgenic expression of the human NAD-hydrolyzing ectoenzyme CD38 inhibits plant immunity. We therefore hypothesize that, during plant–microbe interactions, NAD is released from dead or dying cells into the extracellular space where it interacts with adjacent naïve cells’ surface receptors, which in turn activate downstream immune signaling. However, it is currently unknown whether eNAD signaling is unique to *Arabidopsis* or the Brassicaceae family. In this study, we treated citrus plants with exogenous NAD^+^ and tested NAD^+^-induced transcriptional changes and disease resistance. Our results show that NAD^+^ induces profound transcriptome changes and strong resistance to citrus canker, a serious citrus disease caused by the bacterial pathogen *Xanthomonas citri* subsp. *citri* (*Xcc*). Furthermore, NAD^+^-induced resistance persists in new flushes emerging after removal of the tissues previously treated with NAD^+^. Finally, NAD^+^ treatment primes citrus tissues, resulting in a faster and stronger induction of multiple salicylic acid pathway genes upon subsequent *Xcc* infection. Taken together, these results indicate that exogenous NAD^+^ is able to induce immune responses in citrus and suggest that eNAD may also be an elicitor in this woody plant species.

## Introduction

Nicotinamide adenine dinucleotide (NAD) is a ubiquitous electron carrier that functions in both metabolic reactions and signaling events inside the cell (Berger et al., 2004; Noctor et al., 2006). Upon environmental stimuli, cellular NAD can also be actively or passively released into the extracellular space, where it is processed or perceived by ectoenzymes or cell-surface receptors, resulting in transmembrane signaling (Bruzzone et al., 2001; Contreras et al., 2003; Seman et al., 2003; Adriouch et al., 2007). In recent years, accumulating evidence indicates that extracellular NAD (eNAD) plays a unique signaling function in diverse physiological and pathological processes (Billington et al., 2006; Haag et al., 2007; Adriouch et al., 2012).

In animal cells, two groups of eNAD processing ectoenzymes, CD38/CD157 and mono(ADP-ribosyl)transferases (ARTs), have been well characterized (Billington et al., 2006). CD38 is a multifunctional ectoenzyme that utilizes NAD as the substrate to produce cyclic ADP-ribose, a second messenger triggering calcium release from intracellular stores (Ceni et al., 2003; De Flora et al., 2004; Krebs et al., 2005; Malavasi et al., 2006; Morabito et al., 2006; Partida-Sanchez et al., 2007). ARTs are another group of ectoenzymes, which use NAD as the substrate to ADP-ribosylate lipid raft-associated proteins (Nemoto et al., 1996; Han et al., 2000; Seman et al., 2003; Bannas et al., 2005; Zolkiewska, 2005). eNAD seems also to be perceived by cell-surface receptors (Moreschi et al., 2006; Mutafova-Yambolieva et al., 2007; Grahnert et al., 2009; Klein et al., 2009). However, a bona fide eNAD-binding receptor has not been identified in animal cells.

In plants, NAD has also been documented to function in response to environmental stresses including pathogen infections (Noctor et al., 2006; Mahalingam et al., 2007; Hashida et al., 2009; Pétriacq et al., 2013; Gakière et al., 2018). For instance, quinolinate-induced elevation of intracellular NAD in *Arabidopsis thaliana* expressing the *nadC* gene from *Escherichia coli*, which encodes the NAD biosynthesis enzyme quinolinate phosphoribosyltransferase, increases defense gene expression and resistance to multiple bacterial and fungal pathogens (Pétriacq et al., 2012, 2016). Conversely, mutations in the NAD biosynthesis gene *FLAGELLIN-INSENSITIVE4* inhibit stomatal immunity in *Arabidopsis*. Moreover, overexpressing the *A. thaliana* *Nudix hydrolase homolog6* (*AtNUDT6*), which encodes an ADP-ribose/NADH pyrophosphohydrolase, and knocking out *AtNUDT6*, *AtNUDT7*, or *AtNUDT8* all lead to changes in intracellular NADH levels and salicylic acid (SA)-mediated immune signaling (Bartsch et al., 2006; Ge et al., 2007; Ishikawa et al., 2010; Fonseca and Dong, 2014).

We have recently shown in the model plant *Arabidopsis* that exogenous NAD induces SA-dependent and -independent expression of *PATHOGENESIS-RELATED* (*PR*) genes and resistance to the bacterial pathogen *Pseudomonas syringae* (Zhang and Mou, 2009, Mou, 2012). Moreover, we discovered that *P. syringae*-induced cell death leads to release of NAD into the extracellular space at concentrations sufficient for induction of *PR* gene expression and *P. syringae* resistance (Zhang and Mou, 2009). In addition, we found that expression of the human NAD-metabolizing ectoenzyme CD38 inhibits biological induction of systemic acquired resistance (SAR), a long-lasting form of plant immunity (Zhang and Mou, 2012). These results together suggest that eNAD may be a damage-associated molecular pattern (DAMP) in plants (Mou, 2017).

We subsequently used both forward and reverse genetic approaches to establish the eNAD signaling pathway in *Arabidopsis*. In the forward genetic screen, we revealed that the mediator complex subunits MED14/STRUWWELPETER and MED16/SENSITIVE TO FREEZING6/INSENSITIVE TO EXOGENOUS NAD1 as well as the elongator complex are downstream signaling components in the eNAD signaling pathway (Zhang et al., 2012, 2013; An et al., 2016). In the reverse genetic screen, we demonstrated that the lectin receptor kinase (LecRK), LecRK-I.8, is an eNAD-binding receptor and functions in plant basal immunity (Wang et al., 2017). These findings support that eNAD is a DAMP in *Arabidopsis*. However, it is not clear whether eNAD signaling is specific for *Arabidopsis* or the Brassicaceae family (Mou, 2017). In this study, we tested if NAD could activate immune responses in sweet orange (*Citrus sinensis* L. Osbeck), one of the most economically important tree fruit crops in the world. Our results indicate that treatment of citrus plants with exogenous NAD^+^ induces profound transcriptional changes and strong resistance to citrus canker, a serious citrus disease caused by the bacterial pathogen *Xanthomonas citri* subsp. *citri* (*Xcc*) (Graham et al., 2004). These results suggest that eNAD signaling may be conserved in citrus.

## Materials and Methods

### Plant Materials and Growth Conditions

Pineapple sweet orange seedlings were maintained in standard greenhouses at the Citrus Research and Education Center of the University of Florida. Citrus seedlings were cut back to produce expanding leaves 6 weeks prior to NAD^+^ treatments.

### Chemical Treatment

NAD^+^ sodium salt (Sigma-Aldrich, St. Louis, MO, United States) was dissolved in water and pH was adjusted to 6.0 using 0.1 M NaOH. For soil drenches, 7 days in advance of *Xcc* inoculation when seedlings reached 50 to 75% expanded flush, 250 mL NAD^+^ solution (1, 5, or 10 mM) per potted seedling were applied. For infiltration, 1 day in advance of *Xcc* inoculation leaves were infiltrated with various concentrations of NAD^+^ (0, 0.25, 0.5, 0.75, 1, 5, and 10 mM) using a needleless tuberculin syringe (1.0 mL) as previously described (Graham and Leite, 2004). For the positive control, Actigard (Syngenta, Greensboro, NC, United States) was dissolved in water (2 g/L) and 250 mL of the resulting solution was applied to each potted citrus seedling by soil drenching.

### Pathogen Infection

Five plants per treatment were inoculated by injection-infiltration with a suspension of 10^4^ cfu/mL of *Xcc* strain X2002-0014 in PBS by pressing the needleless syringe tip against the leaf surface to produce a zone of water-soaked tissue in three areas on each side of the midrib to produce six distinct inoculation sites per leaf on three to four leaves per replicate plant for a total of 15 leaves per treatment. Inoculated shoots were covered with plastic bags for 1 day to maintain high humidity conducive for bacterial infection of leaves. At 14 day post-inoculation, lesions were counted at each inoculation site and summed as total lesions per leaf.

To determine the persistence of the treatment effect over time (previously called residual activity) (Francis et al., 2009), plants were pruned below the *Xcc* inoculation point (6 weeks after NAD application). When new single shoots produced four to six young leaves, a new set of *Xcc* inoculations were performed (16 weeks after NAD applications).

### RNA Analysis

Total RNA extraction, reverse transcription, and real-time quantitative PCR (qPCR) were performed as previously described (Llorens et al., 2015) with some modifications. Briefly, six disks of 6-mm diameter (one per inoculation site) from three leaves per plant from at least three plants per biological replicates were collected per each time point and frozen in liquid nitrogen, and tissues were ground twice at 30 rps in a Tissuelyzer II (QIAGEN, Hilden, Germany). RNA was extracted using an RNeasy Mini kit (QIAGEN) according to the manufacturer’s instructions and RNA concentration was determined using a ND-1000 spectrophotometer (NanoDrop Technologies, Wilmington, DE, United States). One microgram of total RNA was used for cDNA synthesis with a QuantiTect Reverse Transcription kit (QIAGEN) according to the manufacturer’s instructions. qPCR was performed on a 7500 Fast Real-Time PCR System (Applied Biosystems, Foster City, CA, United States) using a QuantiTect SYBR Green PCR kit (QIAGEN) in 20 μL reactions following the manufacturer’s instructions. Samples were run in triplicate. The occurrence of non-specific amplification products was ruled out after performing a melting curve analysis. The relative gene expression analysis was performed using the ΔCt method. Time 0 expression was arbitrarily set as 1 and then data of each time point were normalized against time 0. Results were presented as mean ± standard error of three biological samples run in triplicate. Primers used in this study were listed in **Supplementary Table S2**.

### Microarray Analysis

Microarray analysis using the Affymetrix microarray platform was performed at the University of Florida Interdisciplinary Center for Biotechnology Research. RNA quality was assessed using the Agilent 2100 Bioanalyzer (Agilent Technologies, Inc., Santa Clara, CA, United States). All microarray sample preparation used the GeneChip^®^ 3^′^ IVT Plus Express kit (Affymetrix, Inc., Santa Clara, CA, United States), and reactions were done following the manufacturer’s protocols. Briefly, cDNA was synthesized from 200 ng of total RNA and template for *in vitro* transcription during which a biotin-modified nucleotide was incorporated. The biotin-labeled aRNA was then purified and fragmented. Samples were hybridized with rotation at 45°C for 16 h to the Affymetrix GeneChip Citrus Genome Array. The arrays were washed and stained with the reagents supplied in GeneChip^®^ Hybridization Wash and Stain kit (Affymetrix, Inc.) on an Affymetrix Fluidics Station 450, and scanned with a GeneChip^®^ 7G Scanner (Affymetrix, Inc.).

The microarray data were pre-processed and normalized using the affy package. The Robust Multichip Analysis approach was applied for the normalization. After normalization, the empirical Bayes moderated *t*-statistics, which is implemented in the limma Bioconductor package (Smyth, 2004), was performed for differential expression detection. In each comparison, a *p*-value and fold change were computed for each gene locus. The gene expression fold changes were computed based on the normalized log-transformed signal intensity data. To control false discovery rate and correct multiple hypothesis testing, a *q*-value was calculated and used to assess the significance of each test using Benjamini and Hochberg’s approach (Benjamini and Hochberg, 1995). Genes with an absolute fold change ≥ 2 and a *q*-value ≤ 0.05 were considered as significantly differentially expressed.

### Statistical Methods

Statistical analyses were performed by one-way ANOVA using SAS (SAS Institute Inc., Cary, NC, United States) followed by a Student–Newman–Keuls test. All experiments were repeated at least three independent times with similar trends. Results from a representative experiment are presented.

## Results

### Exogenous NAD^+^ Induces Resistance to Citrus Canker in a Concentration-Dependent Manner in Citrus

**FIGURE 1:**
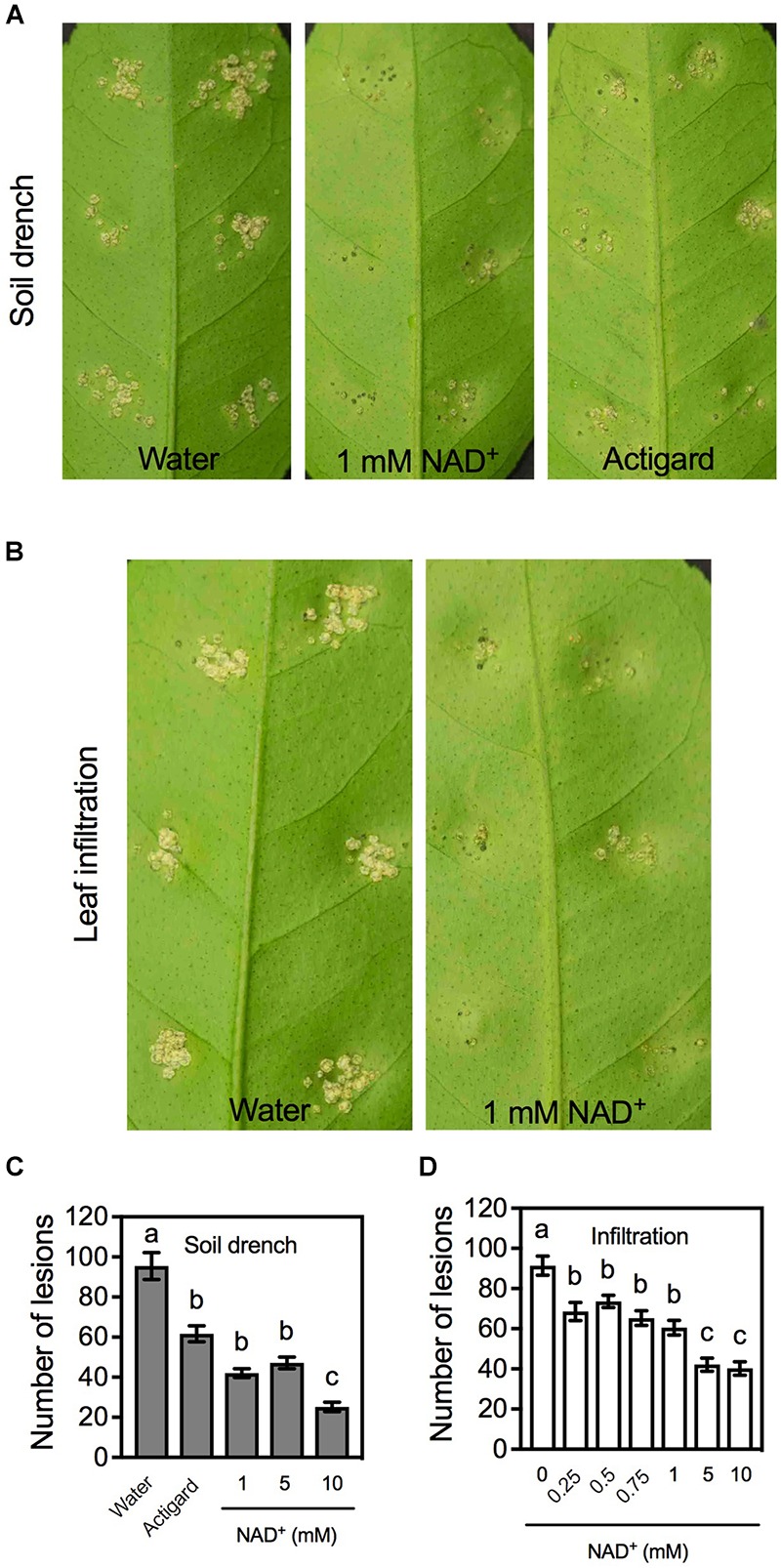
Exogenous NAD^+^-induced resistance to citrus canker in citrus. **(A)** Phenotypes of the citrus canker lesions on leaves of citrus plants treated with soil drenches of water, 1 mM NAD^+^, or Actigard. Photos were taken 14 days after *Xcc* inoculation. **(B)** Phenotypes of the citrus canker lesions on leaves pre-infiltrated with water or 1 mM NAD^+^. Photos were taken 14 days after *Xcc* inoculation. **(C)** Numbers of the citrus canker lesions on leaves of citrus plants treated with soil drenches of water, Actigard, or different concentrations (1, 5, and 10 mM) of NAD^+^. Data represent means of five biological replicates with standard error (SE). Different letters above the bars indicate significant differences (Newman–Keuls test, *p* < 0.05). **(D)** Numbers of the citrus canker lesions on leaves pre-infiltrated with water or various concentrations (0.25, 0.5, 0.75, 1, 5, and 10 mM) of NAD^+^. Data represent means of five biological replicates with SE. Different letters above the bars indicate significant differences (Newman–Keuls test, *p* < 0.05).

To test whether exogenous NAD^+^ induces immune responses in citrus, citrus plants were treated with different concentrations of NAD^+^ by soil drenches or leaf infiltration. It has previously been shown that soaking plus foliar sprays of SA and soil drenches of Actigard, a product containing the SA analog acibenzolar-S-methyl, induce strong and persistent resistance to citrus canker (Francis et al., 2009; Graham and Myers, 2009; Wang and Liu, 2012). We included Actigard in the soil drench experiments as the positive control. After the chemical treatment, leaves on the treated plants or the infiltrated leaves were inoculated with *Xcc*. Symptoms were characterized on day 14 post-inoculation. As shown in **Figure 1A**, leaves on water-treated plants produced callus-like lesions, whereas those on NAD^+^- or Actigard-treated plants developed fewer necrotic lesions that were smaller in size. Similarly, leaves pre-infiltrated with water generated callus-like lesions, whereas those pre-infiltrated with NAD^+^ formed fewer and smaller necrotic lesions (**Figure 1B**). The disease symptoms on the plants drenched with 1mM NAD^+^ and the leaves pre-infiltrated with 1 mM NAD^+^ were comparable to those developed on the plants drenched with Actigard (2 μg/mL) (**Figures 1A,B**).

The resistance induced by NAD^+^ was also reflected by the numbers of lesions developed at the inoculation sites. Overall, the lesion numbers on the plants or leaves treated with Actigard or NAD^+^ were significantly lower than those on the water-treated controls. For soil drenches, the lesion numbers on the plants treated with 1 and 5 mM NAD^+^ were comparable to those on the plants treated with Actigard, whereas the lesion numbers on the plants treated with 10 mM were significantly lower than those on the Actigard-treated plants (**Figure 1C**). For infiltration, the lesion numbers on the leaves treated with 0.25, 0.5, 0.75, and 1 mM NAD^+^ were not significantly different from each other, whereas the lesion numbers on leaves treated with 5 and 10 mM NAD^+^ were significantly lower than those on the leaves treated with lower concentrations of NAD^+^ (**Figure 1D**). Thus, exogenous NAD^+^ induces disease resistance in citrus in a concentration-dependent manner.

### Exogenous NAD^+^ Triggers Profound Transcriptome Changes in Citrus

To understand the molecular events underlying NAD^+^-induced disease resistance in citrus, a microarray experiment was carried out to examine NAD^+^-triggered transcriptional changes in citrus (National Center for Biotechnology Information [NCBI] Gene Expression Omnibus series number GSE113735). Independent samples in triplicate were assayed, and the results were analyzed to identify genes that displayed a twofold or higher induction or suppression with a low *q*-value (≤0.05) in the NAD^+^-treated samples compared with the water-treated control samples. A total of 660 and 574 genes were up- and down-regulated, respectively, at 4 h after the NAD^+^ treatment (**Supplementary Table S1**). The numbers of genes that were up- and down-regulated in citrus are much smaller than those (2,155 and 2,014 genes up- and down-regulated, respectively) in *Arabidopsis* under similar conditions (Wang et al., 2017). These results suggest that citrus may respond more slowly or be less sensitive to exogenous NAD^+^ treatment than *Arabidopsis*. Nevertheless, a large number of defense-related genes, such as *ENHANCED DISEASE SUSCEPTIBILITY1*, *NIM1-INTERACTING PROTEIN2*, *CHORISMATE MUTASE2* (*CM2*), *PATHOGENESIS-RELATED* (*PR*) *GENES TRANSCRIPTIONAL FACTOR PTI5*, *PR4A*, *PR4B*, *GLUTATHIONE S-TRANSFERASE6*, just to name a few, were up-regulated by the NAD^+^ treatment (**Table 1** and highlighted in yellow in **Supplementary Table S1**). Thus, as in *Arabidopsis* (Wang et al., 2017), exogenous NAD^+^ treatment activates defense signaling pathways in citrus.

**Table 1:** Possible defense-related genes that were induced by NAD^+^ treatment.

Probeset ID	NAD^+^/Water		Transcript ID	Target description
	Log2(FC)	*q*-value		
Cit.12743.1.S1_at	3.48	0.011	Cit.12743.1	Putative glutathione *S*-transferase T3
Cit.36935.1.S1_s_at	2.92	0.006	Cit.36935.1	*S*-Adenosyl-L-methionine:salicylic acid carboxyl methyltransferase
Cit.16841.1.S1_at	2.84	0.000	Cit.16841.1	NIM-interacting protein 2 (NIMIN2)
Cit.2730.1.S1_at	2.11	0.009	Cit.2730.1	Mitogen-activated protein kinase
Cit.9675.1.S1_at	1.78	0.000	Cit.9675.1	Glutathione transferase
Cit.14156.1.S1_s_at	1.76	0.008	Cit.14156.1	Mitogen-activated protein kinase kinase (MAPKK) 9, putative (MKK9)
Cit.36833.1.S1_at	1.70	0.003	Cit.36833.1	MATE efflux family protein
Cit.16865.1.S1_at	1.61	0.004	Cit.16865.1	Thioredoxin H
Cit.753.1.S1_x_at	1.58	0.015	Cit.753.1.S1	Pathogenesis-related protein 4A (PR4A)
Cit.2113.1.S1_at	1.58	0.000	Cit.2113.1	Disease resistance-responsive protein-related/dirigent protein-related
Cit.21717.1.S1_at	1.51	0.001	Cit.21717.1	Pathogenesis-related protein 4b (PR4B)
Cit.9293.1.S1_at	1.48	0.003	Cit.9293.1	UDP-glucose:salicylic acid glucosyltransferase
Cit.2058.1.S1_s_at	1.44	0.000	Cit.2058.1	MATE efflux family protein
Cit.17342.1.S1_at	1.43	0.003	Cit.17342.1	Chorismate mutase, cytosolic (CM2)
Cit.18086.1.S1_s_at	1.42	0.027	Cit.18086.1	Pathogenesis-related genes transcriptional activator PTI5
Cit.19571.1.S1_s_at	1.40	0.010	Cit.19571.1	Disease resistance-responsive protein-related/dirigent protein-related
Cit.29252.1.S1_at	1.29	0.048	Cit.29252.1	EDS1
Cit.6165.1.S1_at	1.26	0.004	Cit.6165.1	Putative MATE efflux protein family protein
Cit.25547.1.S1_at	1.26	0.000	Cit.25547.1	Leucine-rich repeat receptor-like kinase
Cit.29940.1.S1_at	1.25	0.000	Cit.29940.1	Protein phosphatase 2C-like protein
Cit.25902.1.S1_at	1.24	0.010	Cit.25902.1	Glutathione *S*-transferase 6 (GST6)
Cit.17645.1.S1_x_at	1.19	0.000	Cit.17645.1	Glutathione *S-*transferase
Cit.3694.1.S1_at	1.17	0.001	Cit.3694.1	Putative protein phosphatase 2C
Cit.8664.1.S1_x_at	1.17	0.000	Cit.8664.1	MATE efflux family protein
Cit.2116.1.S1_s_at	1.17	0.024	Cit.2116.1	Pathogenesis-related protein 5-1
Cit.17228.1.S1_x_at	1.16	0.022	Cit.17228.1	Immediate-early salicylate-induced glucosyltransferase pir
Cit.8482.1.S1_x-at	1.16	0.000	Cit.8482.1	Glutathione *S*-transferase
Cit.4233.1.S1_s_at	1.13	0.004	Cit.4233.1	Disease resistance protein, putative
Cit.34261.1.S1_s_at	1.12	0.000	Cit.34261.1	MATE efflux family protein
Cit.23245.1.S1_at	1.09	0.007	Cit.23245.1	Glutathione *S*-transferase
Cit.18055.1.S1_s_at	1.09	0.019	Cit.18055.1	Putative leucine-rich repeat receptor kinase
Cit.356.1.S1_s_at	1.07	0.004	Cit.356.1	Thioredoxin H
Cit.6308.1.S1_at	1.07	0.037	Cit.6308.1	Putative glutathione *S*-transferase T3
Cit.6136.1.S1_at	1.04	0.003	Cit.6136.1	Bacterial spot disease resistance protein 4
Cit.28421.1.S1_s_at	1.03	0.005	Cit.28421.1	Putative cyclic nucleotide and calmodulin-regulated ion channel protein
Cit.26653.1.S1_at	1.01	0.003	Cit.26653.1	EIX receptor 2


### Exogenous NAD^+^ Activates Long-Lasting Resistance in Citrus

**FIGURE 2:**
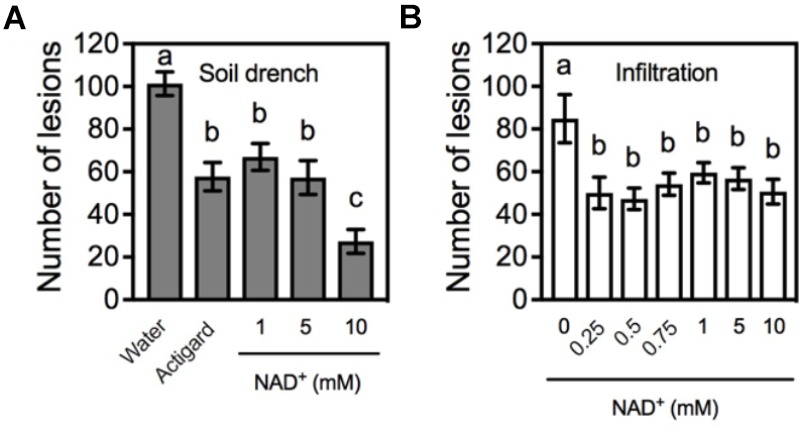
The effectiveness of NAD^+^ treatment over time. **(A)** Numbers of the citrus canker lesions on leaves of new shoots emerged on the citrus plants that were previously treated with soil drenches of water, Actigard, or different concentrations (1, 5, and 10 mM) of NAD^+^. The plants were pruned below the inoculation point 6 weeks after NAD^+^ treatment. Data represent means of five biological replicates with SE. Different letters above the bars indicate significant differences (Newman–Keuls test, *p* < 0.05). **(B)** Numbers of the citrus canker lesions on leaves of new shoots emerged on the citrus plants with leaves previously infiltrated with water or various concentrations (0.25, 0.5, 0.75, 1, 5, and 10 mM) of NAD^+^ and inoculated with *Xcc*. The plants were pruned below the inoculation point 6 weeks after NAD^+^ treatment. Data represent means of five biological replicates with SE. Different letters above the bars indicate significant differences (Newman–Keuls test, *p* < 0.05).

Previous work has shown that the effectiveness of soil-applied Actigard can last for more than 16 weeks (Francis et al., 2009). To determine the effectiveness of soil-applied NAD^+^ over time, plants were pruned below the *Xcc* inoculation point (6 weeks after NAD^+^ treatment). When new single shoots produced four to six young leaves, the young leaves were inoculated with *Xcc* (16 weeks after NAD^+^ treatment). On day 14 post-inoculation, lesions developed on the inoculated leaves were counted. As shown in **Figure 2A**, the effectiveness of soil drenches of 1 or 5 mM NAD^+^ was comparable to that of Actigard, whereas 10 mM NAD^+^ had a stronger effect at this time point. We also tested the effectiveness of leaf infiltration of NAD^+^ over time. Intriguingly, leaf infiltration of NAD was also able to provide protection to the newly emerged tissues against citrus canker, and the effectiveness was comparable among the applied six different concentrations (0.25, 0.5, 0.75, 1, 5, and 10 mM) (**Figure 2B**).

### Exogenous NAD^+^ Primes Citrus Leaf Tissues

**FIGURE 3:**
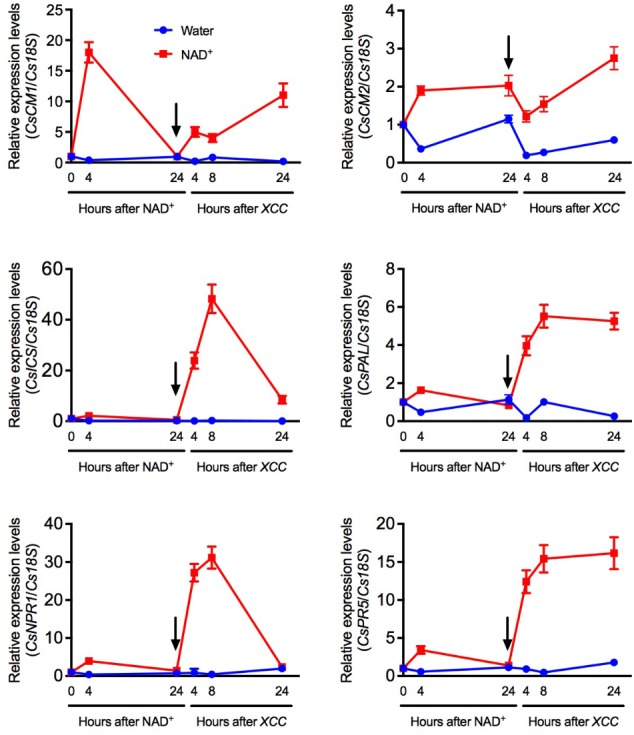
NAD^+^-mediated priming of several SA-related defense genes. Expression of *CsCM2*, *CsCM1*, *CsICS*, *CsPAL*, *CsNPR1*, and *CsPR5* at the indicated time points in citrus leaf tissues first infiltrated with 5 mM NAD^+^ and then inoculated with *Xcc*. The arrows indicate the time point when the leaf tissues were inoculated with *Xcc* after NAD^+^ treatment. Expression of the target genes was normalized against the constitutively expressed *Cs18S*. The *y*-axis values represent the relative expression levels of the indicated genes, which were calculated using the formula 2^[Ct(^*^Cs18S^*^)-Ct(targetgene)]^. Data represent means of three biological replicates with SE.

The long-lasting resistance provided by NAD^+^ treatment might be due to NAD’s priming effects. To test this hypothesis, we infiltrated citrus leaves with NAD^+^ and half of the infiltrated leaves were collected at 4 and 24 h, and the other half of the NAD^+^-infiltrated leaves were inoculated with *Xcc* at 24 h after NAD^+^ treatment. Expression of six SA pathway genes, *CsCM2*, *CsCM1*, *CsICS* (*ISOCHORISMATE SYNTHASE*), *CsPAL* (*PHENYLALANINE AMMONIA LYASE*), *CsNPR1* (*NON-EXPRESSOR OF PR GENES*), and *CsPR5*, was analyzed by qPCR. At 4 h after NAD^+^ treatment, *CsCM2* and *CsCM1* were dramatically induced, *CsPAL*, *CsNPR1*, and *CsPR5* were slightly induced, and *CsICS* was barely induced (**Figure 3**). Surprisingly, after *Xcc* inoculation, all six genes were induced to much higher levels in the leaf tissues pre-treated with NAD^+^ than in those pre-treated with water (**Figure 3**). These results indicate exogenous NAD^+^ treatment primes these SA pathway genes.

## Discussion

Although eNAD is being established as a novel DAMP in *Arabidopsis*, its role in other plant species has not been investigated (Mou, 2017). In this study, we tested exogenous NAD^+^-triggered immune responses in citrus. Our results show that exogenous NAD^+^ induced strong resistance to citrus canker (**Figures 1A–D**), a serious leaf and fruit disease damaging multiple economically important citrus cultivars including grapefruit and certain sweet oranges (Graham et al., 2004). Furthermore, NAD^+^ treatment triggered profound transcriptome changes in citrus leaves, with about 1,200 genes being up-regulated or down-regulated by twofold or more (**Table 1** and **Supplementary Table S1**). These results indicate that citrus leaf tissues are highly responsive to exogenous NAD^+^ treatment. Thus, as the herbaceous plant *Arabidopsis* (Zhang and Mou, 2009; Wang et al., 2017), the woody plant citrus may also possess sensitive eNAD perception mechanisms.

Exogenous NAD^+^ provides long-lasting protection against citrus canker in citrus. Previous work showed that soil drenches of the SAR-inducing product Actigard was able to protect against citrus canker for 16–24 weeks (Francis et al., 2009). The effect of soil drenches of NAD^+^ was comparable to that of Actigard, which in our experiments lasted for at least 16 weeks (**Figure 2A**). This long-lasting protection may be attributed to the priming effect of NAD^+^ on defense genes such as the SA pathway genes tested in **Figure 3**. Our previous work in *Arabidopsis* has also shown that although low concentrations (0.2 and 0.4 mM) of NAD^+^ did not drastically induce *PR* gene expression, they significantly enhanced resistance to the bacterial pathogen *P. syringae* pv. *maculicola* ES4326 (Zhang and Mou, 2009). It is thus likely that low concentrations of NAD^+^ are also sufficient for priming defense genes in citrus (**Figure 2B**).

Surprisingly, resistance induced by leaf infiltration of NAD^+^ was also able to persist in newly emerged shoots even after removal of the NAD^+^-treated leaves together with the shoots (**Figure 2B**). It is possible that NAD^+^, its derivatives, or NAD^+^-induced signaling molecules are able to move throughout the citrus plants, leading to resistance in new flushes. In agreement with this observation, we have shown in *Arabidopsis* that local infiltration of 5 mM NAD^+^ or NADH, a concentration higher than the physiological levels, was able to induce resistance in systemic leaves (Zhang and Mou, 2012). Although these results suggest that NAD may induce SAR, further investigations are required to fully understand if and how endogenous eNAD activates immunity systemically in plants.

## Author Contributions

ZM and JG conceived and designed the experiments. FA and KG performed the experiments. FA, J-LL, and YZ analyzed the data. ZM and FA wrote the paper. All the authors carefully checked and approved this version of the manuscript.

## Conflict of Interest Statement

The authors declare that the research was conducted in the absence of any commercial or financial relationships that could be construed as a potential conflict of interest.
