# Supplementary material for: Exogenous Nicotinamide Adenine Dinucleotide Induces Resistance to Citrus Canker in Citrus
Source: Front Plant Sci. 2018 Oct 9;9:1472. doi: 10.3389/fpls.2018.01472 (PMC6189366; doi:10.3389/fpls.2018.01472)
Supplement: Supplementary file 2 [file Table_2.DOCX]

**Table S2. Primers used for qPCR in this study**

| Gene | Forward primer (5’-3’) | Reverse primer (5’-3’) |
| --- | --- | --- |
| *CsCM2* (Cs7g06370) | CCTGGCTTCTCTGGTTCTTT | GAAGGGACTTTCTTCTGGATTCT |
| *CsCM1* (Cs6g21940) | CATGAAACTGGAGGGCATAAGA | CGTTGTCCGTCTATGGTGAAA |
| *CsICS* (Cs5g04210) | GGAGGAGGAGAGAGTGAATTTG | GGGTTGCTTCCTTCTACTATCC |
| *CsPAL* (Cs6g11940) | CACATTCTTGGTAGCGCTTTG | AGCTACTTGGCTGACAGTATTC |
| *CsNPR1* (Cs4g14600) | GTACCTTGAAAACAGAGTTGGACTGG | TGCTCCTCTTGCATTTTGAAAGGTG |
| *CsPR5* (Cs8g02870) | TACCTCCACCTCTCTCATTCTT | GTGCGAGAGAAGGTTAGCTATG |
| *Cs18S* | GTGACGGAGAATTAGGGTTCG | CTGCCTTCCTTGGATGTGGTA |
